# Bayes' Theorem to estimate population prevalence from Alcohol Use Disorders Identification Test (AUDIT) scores

**DOI:** 10.1111/j.1360-0443.2009.02574.x

**Published:** 2009-07

**Authors:** David R Foxcroft, Kypros Kypri, Vanessa Simonite

**Affiliations:** 1 Oxford Brookes UniversityOxford, UK; 2 University of NewcastleNewcastle, Australia

**Keywords:** Alcohol drinking, alcohol use disorders, alcoholism, alcohol-related disorders, Bayes' theorem, epidemiology

## Abstract

**Aim:**

The aim in this methodological paper is to demonstrate, using Bayes' Theorem, an approach to estimating the difference in prevalence of a disorder in two groups whose test scores are obtained, illustrated with data from a college student trial where 12-month outcomes are reported for the Alcohol Use Disorders Identification Test (AUDIT).

**Method:**

Using known population prevalence as a background probability and diagnostic accuracy information for the AUDIT scale, we calculated the post-test probability of alcohol abuse or dependence for study participants. The difference in post-test probability between the study intervention and control groups indicates the effectiveness of the intervention to reduce alcohol use disorder rates.

**Findings:**

In the illustrative analysis, at 12-month follow-up there was a mean AUDIT score difference of 2.2 points between the intervention and control groups: an effect size of unclear policy relevance. Using Bayes' Theorem, the post-test probability mean difference between the two groups was 9% (95% confidence interval 3–14%). Interpreted as a prevalence reduction, this is evaluated more easily by policy makers and clinicians.

**Conclusion:**

Important information on the probable differences in real world prevalence and impact of prevention and treatment programmes can be produced by applying Bayes' Theorem to studies where diagnostic outcome measures are used. However, the usefulness of this approach relies upon good information on the accuracy of such diagnostic measures for target conditions.

## INTRODUCTION

In epidemiological and clinical research surrogate indicators are often used for convenience: either because the actual outcomes are too far in the future or because they are too expensive or difficult to measure directly. Examples of common surrogate indicators include blood pressure, body mass index and blood glucose levels. Surrogate indicators are more or less accurate and sometimes may not reflect the true outcome of interest usefully. For example, in the Cardiac Arrhythmia Suppression Trial [1] many therapies were approved using an agreed surrogate indicator which was responsive to therapy, namely the frequency and complexity of premature ventricular contractions. However, the longer-term trial results showed up the poor accuracy of the surrogate indicator when the actual outcome of arrhythmic sudden death was considered. In this paper we describe an approach to improve the interpretation and utility of trials using surrogate indicators.

Self-report questionnaire scales are often used as surrogate indicators of health outcomes from treatment and prevention interventions. Where pre-existing evidence of the accuracy of self-report questionnaire scales against a positive diagnosis, or a gold standard measurement, is available then information about the accuracy of the surrogate indicator is also present. For example, the Alcohol Use Disorders Identification Test (AUDIT) or similar tools provide diagnostic information that can be useful in research and in practice, for example in screening and brief intervention (SBI) programmes for alcohol misuse. Typically a cut-off score, or threshold, is used to rule someone in, or out, by way of a tentative diagnosis. For AUDIT, a conventional threshold for individuals at higher risk of alcohol problems is a score of 8 or more [2].

While this approach may have advantages in practice settings, there are disadvantages if a simple threshold approach is used in epidemiological research or policy work where questions focus upon population prevalence and effectiveness of interventions. Each point, or score, on a diagnostic scale provides useful information that may be lost if scores are collapsed together into negative or positive categories. However, studies of such diagnostic tests often simplify their results by calculating sensitivity and specificity when compared to a gold standard criterion for the presence or absence of a condition or disease, where all values above a single threshold level are considered ‘positive’ and all those below it are considered ‘negative’. This simplistic approach implies that all test results above the threshold increase the likelihood that the condition or disease is present to exactly the same degree. However, if the likelihood associated with a range of different thresholds, for example each point on a diagnostic test scale, can be calculated then much more precise estimates of the risk of a condition or disease can be made. This is particularly important when risk increases proportionately or exponentially with test score.

Similarly, in randomized controlled trials the difference in diagnostic test scale mean scores between an intervention group and a control group, when measured at follow-up, is interpreted typically as the effect of the intervention. However, this interpretation is often not straightforward because the scales used are typically abstract measures with an uncertain relationship to diagnostic information. For example, what is the relevance of a two-point average reduction on a problem drinking scale such as AUDIT? Is this an important difference in terms of population drinking problems, and therefore policy relevant, or is it a trivial effect of no policy relevance at all?

An illustration of this interpretation challenge can be seen in results from a randomized controlled trial of a screening and brief intervention programme among college student drinkers in New Zealand [3]. These results are reported in terms of change in mean score on the 10-item AUDIT scale. Each item on the AUDIT scale is scored 0–4, giving an overall possible score range of 0–40, with a higher score indicating heavier drinking. In this study the difference in AUDIT score associated with the intervention was 2.2; this was the difference between intervention (mean = 12.39) and control (mean = 14.59) groups at 12-month follow-up; but how should this effect be interpreted? What is the potential impact of this reduction in AUDIT score on alcohol disorders in the population, especially when the effect of the intervention is a 2.2-point reduction in average AUDIT score to 12.39 but the scale range is 40 points and the threshold for hazardous drinking is held typically to be an AUDIT score of 8?

In this paper we outline a methodological approach to estimating the potential ‘real world’ impact of a change in levels of problem drinking scores associated with an intervention, illustrated using AUDIT score data from a New Zealand college student study [3]. This methodological approach applies Bayes' Theorem [4] to provide an estimate of the prevalence reduction in alcohol disorders associated with the intervention.

There are several mathematical formulas related to Bayes' Theorem, but they generally boil down to this:


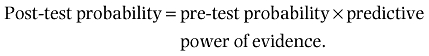


In other words, what we know given the evidence is a function of what we knew even without the evidence, and how good that evidence is.

Suppose an individual has obtained a certain score on a diagnostic test for a condition. What is the probability that the person actually has the condition? It depends upon the pre-test (prior or background) probability of the condition and the accuracy of the test in terms of the known proportions of people with this test score who do and do not actually have the condition. The pre-test probability, for example the general population prevalence or base rate of the condition, can be assumed to be the probability of having the condition when nothing else is known.

If information is available on the pre-test rate of the condition and on the proportion of people at each level of test score having/not having the condition, then Bayes' theorem,


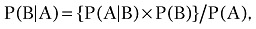


where B means ‘has the condition’ and A means ‘has this score’ may be used to derive, for each person, the post-test (posterior) probability of their having the condition given their score.

## METHOD

We took several steps to develop a model of the prevalence of alcohol abuse or dependence at 12-month follow-up in the New Zealand college student sample, according to study group. First, we obtained a pre-test probability estimate. The pre-test probability can be assumed to be the general population prevalence of a condition, in the absence of any further information. The general population prevalence for alcohol abuse or dependence in this age group (16–24 years) in New Zealand is 7.1%, with 95% confidence interval of 5.7–8.9% [5], based on version 15 of the Composite International Diagnostic Interview (CIDI) for DSM-IV [6], providing a pre-test probability estimate.

The second step was to obtain diagnostic accuracy information for the AUDIT scale in a similar population. Unfortunately, we could not access information for New Zealand so used instead information from a US study [7] of the performance of the AUDIT test against a gold standard in a sample of 358 young people attending a sexually transmitted disease clinic (Dr R. Cook, University of Pittsburgh, personal communication, 2006). In this study 119 young people had a positive alcohol disorder diagnosis based on the gold standard Structured Clinical Interview for DSM-IV (SCID) [8]. From this information we were able to identify the number of people who had a gold standard positive diagnosis (i.e. they had the disorder) and the number of people with a gold standard negative diagnosis (i.e. they did not have the disorder) for each score between 1 and 30 on the AUDIT scale. We labelled these ‘observed N positive’ and ‘observed N negative’.

**Figure 1:**
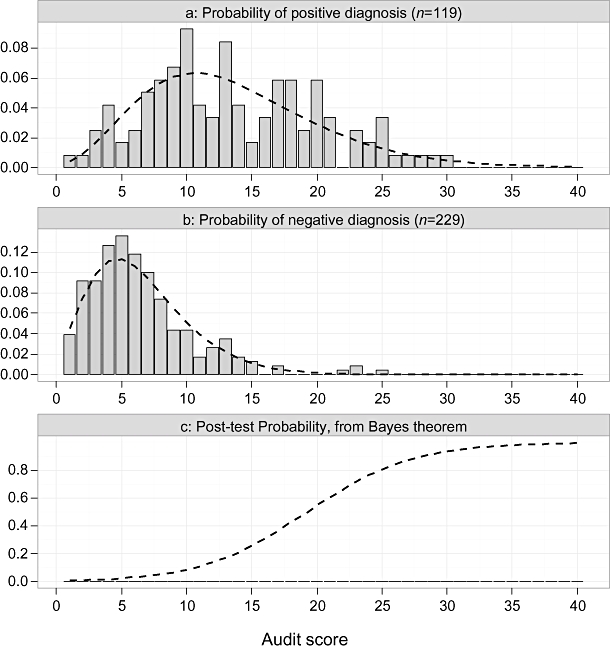
Plot of observed and estimated probability, by Alcohol Use Disorders Identification Test score, of alcohol abuse or dependence, for individuals with a positive diagnosis (a) and negative diagnosis (b); and post-test probabilities using Bayes' Theorem (c; diagnostic data from US study [7] and pre-test probability from NZ national prevalence study [5])

Next, we identified the most appropriate fit function, or curve, for both the observed N positive and the observed N negative distributions from the US data set. We employed this because the observed data was sparse at the higher end of the AUDIT scale and non-existent for scores over 30, and because we required information across the full range of the AUDIT scale. Note that fuller information on the diagnostic properties of the AUDIT scale would obviate this step. For both the observed N positive and the observed N negative distributions we selected a negative binomial function to fit the data because of the discrete properties of the scale (Fig. 1a,b). We used the fit functions to calculate a simulated N positive and a simulated N negative for each AUDIT score and used these along with the pre-test probability to calculate, using Bayes' Theorem, the post-test probability of alcohol abuse or dependence for each AUDIT score between 1 and 40 (Fig. 1c).

Standard errors for the observed N positive and observed N negative were estimated using a bootstrap technique to generate many sets of simulated data to which the negative binomial function was fitted and the standard error calculated for the simulated N positive and the simulated N negative for each AUDIT score. These standard errors, along with the pre-test probability standard error, were propagated through the Bayes' Theorem calculation to provide a standard error estimate for each AUDIT score post-test probability [9].

Finally, working with the NZ data set we entered as new variables each participant's post-test probability corresponding to their AUDIT score at baseline and at 12-month follow-up. So, if an individual had an AUDIT score of 24 at baseline and 18 at follow-up, then their post-test probabilities were specified as 0.76 and 0.42, respectively (Fig. 1c). Averaged post-test probabilities provided an estimate of the prevalence of alcohol abuse or dependence in this sample according to study group and time-point, i.e. the mean post-test probability (prevalence estimate) was calculated for the intervention and control group at baseline and at 12-month follow-up. Standard errors for the prevalence estimates were calculated by combining the standard errors in estimated mean AUDIT scores for the NZ sample and the estimated post-test probabilities of alcohol abuse or dependence for given AUDIT test scores.

All analyses and modelling were undertaken with the R language for statistical computing [10–12].

## RESULTS

**Figure 2:**
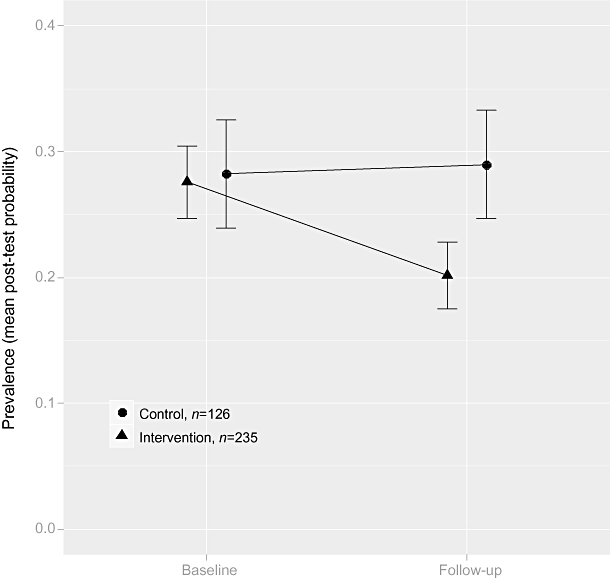
Plot of prevalence (mean post-test probability) and 95% confidence intervals (from propagated standard errors) for alcohol abuse or dependence: NZ randomized controlled trial of web-based social normative intervention

Figure 2 shows the results of this illustrative analysis. Using the NZ study data and AUDIT accuracy information from the US study, using Bayes' Theorem we have estimated that the average post-test probability in the intervention group is 0.20 (95% confidence interval 0.17–0.23) and the average post-test probability in the control group is 0.29 (95% confidence interval 0.25–0.33), providing an estimated population prevalence reduction associated with the SBI intervention of 0.09 (difference between the intervention and control group at 12-month follow-up). The combined standard error for each study group and time-point, based on propagated error terms [9], were used to calculate the 95% confidence interval around the average post-test probabilities, also shown in Fig. 2.

The difference in post-test probability between intervention and control groups at follow-up is analogous to an absolute risk reduction of 9% with 95% confidence interval of 3–14% [9]. This interpretation of the study results is evaluated more easily by policy makers and clinicians than a 2.2 reduction in average AUDIT score, and moreover can be specified in terms of the probable number of cases prevented in a particular setting, as follows.

In New Zealand there were 173 000 under-24-year-old undergraduate students enrolled in degree and subdegree programmes in tertiary education in 2005. Of these, based on the NZ study by Kypri *et al.*[3], 59% are likely to be eligible for a brief intervention programme (e.g. SBI) based on a positive screen, and we assumed an SBI participation rate of 50% [3]. Applying the estimated absolute risk reduction rate suggests that 4271 cases of alcohol abuse or dependence may have been prevented in this cohort if this SBI programme were rolled out across New Zealand. This estimate is, of course, based on all students rather than new students per year. Dividing this estimate by 3 provides a figure of 1424 cases prevented per new student intake, assuming that undergraduate programmes are 3 years long (with 95% confidence intervals 657–2135). This level of impact is clearly important. Of course, it might be that if the SBI programme were repeated each year then maintained or additional effects could be achieved. Alternatively, students may develop a tolerance to the intervention if it is repeated too often.

## DISCUSSION

Providing other researchers and policy makers with information about potential impact on population prevalence rates is more meaningful than information about changes in test scores. This paper demonstrates how this can be achieved using Bayes' Theorem to calculate the expected change in prevalence associated with estimated changes in diagnostic test scores in an epidemiological research study.

Diagnostic accuracy information is valuable in general epidemiological terms as it can be used to provide prevalence estimates from test scores, although the extent to which robust diagnostic accuracy information is readily available, for a specific population, is not clear. In our example this information was crucial in allowing us to estimate, for each AUDIT test score, the post-test probability of alcohol abuse or dependence. Our illustration used data from studies in New Zealand and the United States, i.e. from different settings and countries, hence the results presented in this paper should not be regarded as conclusive: the validity of the diagnostic information from the US study for a NZ college student population is not clear. Furthermore, the accuracy of AUDIT scores as an indicator of alcohol dependence in college students has been questioned [13]. There is also a need to investigate whether such tests perform in the same way in a post-intervention group, i.e. whether the intervention interacts in some way with test response.

Although we have explained the general approach in this illustrative analysis, it will be important to address issues of accuracy in applications of the technique. For example, more robust diagnostic accuracy information, especially at higher levels of the AUDIT scale, might produce a better and more appropriate fit than the negative binomial function used in this analysis, which could have an effect on the calculated post-test probabilities. Therefore it is important, as indicated above, that full information is gathered and analysed from all available diagnostic studies of the AUDIT scale to provide a comprehensive assessment of the scale's characteristics, for different populations.

In conclusion, we suggest that the technique holds promise and, given further research that addresses the points mentioned above, more meaningful and useful interpretations of research evidence may be possible in the future. Moreover, while our example showed the application of this technique to the results of a trial, a potentially valuable extension would be for use in sample size calculations when planning further studies, allowing these to focus upon effect sizes expressed in terms of prevalence reduction rather than a change in test scores. A final point is that this technique potentially improves the usefulness of surrogate indicators but, as mentioned in the opening paragraph of this paper, this is no absolute guarantee that any change in a surrogate indicator following an intervention will be reflected in a change in the actual prevalence of a disorder.
